# Evaluation of core decompression outcome in systemic lupus erythematosus with hip osteonecrosis: a retrospective cohort study

**DOI:** 10.1186/s42358-023-00345-9

**Published:** 2024-01-02

**Authors:** Pouya Hadighi, Seyedeh Tahereh Faezi, Seyed Mohammad Javad Mortazavi, Mohsen Rokni, Leila Aghaghazvini, Amir Kasaeian, Mohammad Nejadhosseinian, Hoda Haerian, Hamid Reza Fateh

**Affiliations:** 1 Rheumatology Research Center, Tehran University of Medical Sciences, Tehran, Iran; 2 Joint Reconstruction Research Center, Imam Khomeini Hospital, Tehran University of Medical Sciences, Tehran, Iran; 3 Department of Orthopedic Surgery, Tehran University of Medical Sciences, Tehran, Iran; 4 Department of Immunology, School of Medicine, Tehran University of Medical Sciences, Tehran, Iran; 5 Department of Immunology, University of Social Welfare and Rehabilitation Sciences, Tehran, Iran; 6 Department of Radiology, Shariati Hospital, Tehran University of Medical Sciences, Tehran, Iran; 7 Hematology, Oncology and Stem Cell Transplantation Research Center, Research Institute for Oncology, Hematology and Cell Therapy, Tehran University of Medical Sciences, Tehran, Iran; 8 Digestive Diseases Research Center, Digestive Diseases Research Institute, Tehran University of Medical Sciences, Tehran, Iran; 9 Inflammation Research Center, Tehran University of Medical Sciences, Tehran, Iran; 10 Department of Physical Medicine and Rehabilitation, Shariati Hospital, Tehran University of Medical Sciences, Tehran, Iran

**Keywords:** Anti-phospholipid antibodies, Avascular necrosis, Core decompression, Systemic lupus erythematosus, Osteonecrosis, Total hip arthroplasty

## Abstract

**Background:**

Osteonecrosis is a major cause of morbidity for patients with systemic lupus erythematosus (SLE). Although core decompression is an approved and trusted technique to prevent further joint deterioration, this surgical method seems to be less beneficial for SLE patients. We aimed to evaluate the outcomes of core decompression in SLE patients with primary stages of femoral head osteonecrosis.

**Methods:**

In this study, 23 patients (39 affected hip joints) with osteonecrosis of the femoral head with stage II of the disease, based on the Ficat-Arlet classification system, underwent core decompression. Also, patients demographic characteristics, clinical data, medication history, comorbidities, immunological findings, hip plain radiographs, history of total hip arthroplasty after core decompression, and patients satisfaction with joint function according to the Oxford hip score questionnaire were obtained.

**Results:**

In the study, 53.8% of affected joints showed signs of radiographic deterioration in follow-up imaging. Sixty-one and a half percent (61.5%) of patients had unsatisfactory joint performance. A third (33.3%) of affected hip joints underwent total hip arthroplasty up to 5 years from core decompression. SLE patients with a history of receiving bisphosphonate were 83.2% less dissatisfied with their joint function than patients without a history of bisphosphonate use (*P* < 0.02). Of the 23 studied cases, the mean cumulative dose of prednisolone before and after core decompression surgery was 46.41 mg and 14.74 mg respectively. Besides, one case (2.6%) that had a high anti-phospholipid antibodies level during follow-up did not have any radiographic deterioration, and 9 cases (23.1%) had some degrees of radiographic deterioration.

**Conclusions:**

The patients group that used bis-phosphonate, had a higher level of satisfaction with joint function after core decompression. Patients with high-level anti-phospholipid antibodies are related to a poor prognosis after core decompression.

## Background

Systemic lupus erythematosus (SLE) is a chronic autoimmune systemic and multi-organ disease with several clinical manifestations. It is described by autoantibody production and commonly affects childbearing women (assessment ranges from five to ten women for each man) [1]. It can influence the joints, mucocutaneous, kidney,   hematopoietic cells, lungs, brain, and other organs. There is no cure for SLE, but medical interventions and lifestyle modification can improve patients healthcare quality [2, 3].

In some patients with SLE disease, musculoskeletal complications are severe and result in poor prognostic. One of the major musculoskeletal complications is non-traumatic osteonecrosis of the femoral head (ONFH) [4]. ONFH is an arthropathy with unclear and multifactorial pathogenesis that leads to joint damage and physical disability in the patient [5]. Osteonecrosis majorly affects long-bone epiphyses, the distal end of the tibia, femoral head and condyles, and humeral head and occasionally shows a multifocal distribution [6]. Major precipitating factors are corticosteroid consumption, vasculitis, raynaud's phenomenon, production of inflammatory mediators, fibrinolysis disorders, gene polymorphisms, septic arthritis, antiphospholipid syndrome, and metabolic diseases [7]. Many studies have indicated the increased incidence of SLE-ONFH in recent years, and this disease has become one of the serious causes of disability and poor prognosis [8]. The severity of ONHF induced by corticosteroid consumption is directly related to the dose of corticosteroid. Most cases of osteonecrosis in patients taking glucocorticoids were in high cumulative doses [8, 9]. The highest prescribed levels and continuous doses of glucocorticoids in the treatment process have been described as major risk factors for avascular necrosis (AVN) in these patients [10, 11].

Core decompression (CD) is a surgical approach that affects surgical drilling into the site of destroyed bone near the articular. The CD is one of the major common prophylactic operations with early promising outcomes and a low morbidity rate. The CD is considered to improve the pathologic environment in the hip joint, including edema, elevated intravascular pressure, and vascularity destruction which leads to significant pain relief and improved joint function [12]. The CD has been used in SLE-ONFH to delay joint damage that may necessitate hip joint replacement [13].

Magnetic resonance imaging (MRI) as a gold standard technique with high sensitivity and specificity is used for the early diagnosis of osteonecrosis in the primary stages. The most important function of osteonecrosis staging is helping to choose the proper method of treatment [14]. Recent studies indicated a higher percentage of SLE patients suffering from ONFH. MRI assessment of hips and knees in 72 SLE patients with osteonecrosis taking high doses of corticosteroid showed multifocal distribution in 32 cases (44%). Thus, 92 joints had characteristics of osteonecrosis, with a light predominance of knee involvement [15]. Other investigations indicated that CD surgical treatments were favored in less than 23% of the subjects when compared to other management in stage I and it was not useful in stage II [16]. Maniwa et al., suggested that CD is an effective treatment for both non-steroid-related and steroid-related patients in terms of improving hip joint function except for SLE patients with stage I and stage II ONFH [12]. Prior clinical investigations have obtained contradictory results about the possible relationship between ONFH progression in SLE patients and CD surgical treatments.

Therefore, this study aimed to evaluate the outcomes of CD in SLE patients with primary stages of femoral head osteonecrosis. We also evaluated the outcomes of CD in high-risk SLE patients who had been consuming corticosteroids continuously (and other medications) and positive anti-phospholipid antibodies during the disease progression.

## Methods

### Design and participants

In total, 23 SLE patients (39 affected hip joints) enrolled according to the European League Against Rheumatism (EULAR 2019)/American College of Rheumatology (ACR 2019) classification criteria for SLE at least 1 year and up to 15 years (mean 5.26 years) before CD surgery. We conducted a retrospective cohort study in Imam Khomeini and Shariati hospital, Tehran University of Medical Sciences (TUMS), Tehran, Iran. SLE patients with femoral head osteonecrosis (FICAT stage II) who underwent hip joint core decompression between July 2013 to January 2019 entered the study. The Ficat classification recognized five different stages of bone necrosis from stage 0 to stage IV [17]. All patients underwent CD using the same technique. Patients demographic characteristics, clinical data, comorbidities, immunological findings, follow-up hip plain radiographs, and history of total hip arthroplasty (THA) after CD were obtained via reviewing hospital clinical records and interviewing. Evaluation of recovery or regression status of the affected joints was done by reviewing follow-up hip radiographs based on the Ficat-Arlet classification system [17]. Patients level of satisfaction from CD was evaluated with the Oxford Hip score questionnaire [18]. A score of 30 and above indicated adequate satisfactory joint function and did not require any formal treatment. Also, a score less than 29 indicated unsatisfactory joint function.

All procedures performed in studies involving human participants were by the ethical standards of the institutional and/or national research committee and with the 1964 Helsinki declaration and its later amendments or comparable ethical standards. The study protocol was approved by the ethics committee of TUMS (IR.TUMS.MEDICINE.REC.1398.723), and written consent was obtained from all participants.

### Immunological data collection

For all of the patients that enrolled in this study, the level of anti-phospholipid antibodies (Anti-cardiolipin antibodies IgM & IgG), and anti-ds DNA antibody levels were measured by enzyme-linked immunosorbent assays (ELISA) kits. The reference range for anti-ds DNA antibody was: < 30 IU/mL negative, 30–75 IU/mL borderline, and > 75 IU/mL positive. In our study, positive results for IgG (GPL) and IgM (MPL) anti-cardiolipin antibodies (> 15 GPL, U/ml and/or > 10 MPL, U/ml) were considered abnormal levels (weak positive). Also, for the lupus anticoagulant antibody test, the laboratory collected a sample of blood in a vial and added chemicals to test blood clotting abilities. The test measures the time it takes for a clot to form in seconds. The abnormal results for lupus anticoagulant antibody (LA-APTT > 45 seconds) were considered positively.

Serum C3 and C4 levels were assessed by the immuno-turbidimetric method with a normal range (0.9–1.8 g/L) for C3 and (0.1–0.4 g/L) for C4.

### Statistical analysis

The data were stated as mean ± standard deviation (± SD) and ratios were stated as percentages (%). The SPSS (Version 22.0; USA) was used to compare the differences between groups using the t-student or Mann–Whitney U test. Ratios were evaluated using the Chi-squared test. Logistic regression analysis was performed to analyze the risk factors. Univariate analysis was used to assess the variables related to ONFH. Multivariate logistic regression was used in the independent variables with *P* < 0.05.

## Results

### Clinical features of the SLE patients with ONFH

In total, 23 SLE patients (39 affected hip joints) were assessed at least 1 year and up to 15 years (mean 5.26 years) before CD surgery. The female to male ratio was 22 to 1 and the mean age was 30.1 years. The mean time of follow-up after CD was 3.5 years.

In our study population, sixteen patients (82.1%) had bilateral osteonecrosis of the hip joint and seven patients (17.9%) had unilateral osteonecrosis of the hip joint. Twenty-one cases (53.8%) of these joints showed signs of exacerbation deterioration in radiographic findings on follow-up imaging, and 13 of them underwent THA. Among 39 hip joints, 13 joints (33.3%) required THA. The mean time between CD and THA was 4.97 years. Also, 24 joints (61.5%) had low satisfaction with a joint condition. Thirteen out of 24 joints (54.2%) required THA (Table 1).

**Table 1:** Comparison between core decompression surgery alone and core decompression plus THA surgery based on prognostic factors

Prognostic factors	Core decompression N = 26 (%)	Core decompression and THA, N = 13 (%)	*P* value
Male gender	1 (2.6)	1 (2.6)	0.614
Two-sided	1 (2.6)	12 (30.8)	0.261
Abnormal complement levels	4 (10.3)	6 (15.4)	0.046*
High level of anti-phospholipid antibodies	4 (10.3)	6 (15.4)	0.046*
SLE relapse	7 (17.9)	10 (25.6)	0.005**
Kidney damage	12 (30.8)	9 (23.1)	0.179
Neuro damage	4 (10.3)	1 (2.6)	0.506
Azathioprine	7 (17.9)	11 (28.2)	0.002**
HCQ	20 (51.3)	10 (25.6)	1.000
Dexamethasone	3 (7.7)	1 (2.6)	0.711
Methotrexate	5 (12.8)	4 (10.3)	0.424
Mycophenolate mofetil	5 (12.8)	2 (5.1)	0.768
Aspirin	16 (41)	4 (10.3)	0.077
Statins family	6 (15.4)	3(7.7)	1.000
Bisphosphonates family	11 (28.2)	4 (10.3)	0.487
Smoking(Cigarette)	10 (25.6)	3 (7.7)	0.779
Alcohol	12 (30.1)	1 (2.6)	0.363
Radiotherapy	13 (33.3)	0 (0)	0.999
Chemotherapy	13 (33.3)	0 (0)	0.999
Organ transplantation	13 (33.3)	0 (0)	0.999
Prednisolon pre-op (Median, IQR)	20 (30–75)	32 (30–55)	0.432
Prednisolon pre-op (Median, IQR)	22.5 (18.7–35)	7.5 (0–15)	0.624

SLE: systemic lupus erythmatosus, HCQ: hydroxychloroquine, IQR: interquartile range

### Immunological findings of the SLE patients with ONFH

#### Anti-ds DNA antibody

In the 39 cases studied, the mean recorded anti-ds DNA level was 98.3 IU/ml (minimum 22.1 and maximum 258.1 IU/ml). We evaluated the relative risk of joint exacerbation by radiographic findings according to the anti-ds DNA level. The results indicated that 18 cases with low anti-ds DNA levels (mean; 78.5 IU/ml) had no evidence of exacerbation in favor of radiographic findings in the follow-up imaging. In contrast, 21 cases with high anti-ds DNA levels (mean; 111.7 IU/ml) had radiographic signs of joint deterioration exacerbation in follow-up imaging (Table 2).

**Table 2:** Comparison between joint deterioration in follow-up imaging after core decompression with investigated variables

Radiographic deterioration	Uni-variable logistic regression		Multivariable logistic regression	
	Odds ratio (CI 95%)	*P* value	Odds ratio (CI 95%)	*P* value
Male gender	1.176( 0.068–20.262)	0.911		
Bilateral Hip osteonecrosis	0.850 (0.163–4.426)	0.847		
Abnormal C3, C4 levels	1.400 (0.325–6.027)	0.651		
Abnormal aPL levels	12.750 (1.421–14.400)	0.023*	10.301 (0.930–14.140)	0.047*
SLE relapse	5.687 (1.378–23.480)	0.016*	1.900 (0.241–14.998)	0.542
Kidney damage	3.143 (0.846–11.671)	0.087	2.001 (0.217 -18.435)	0.540
Neuro damage	0.526 (0.078–3.565)	0.511		
Azathioprine	7.000 (1.668–29.384)	0.008**		
HCQ	0.914 (0.204–4.088)	0.907		
Methotrexate	0.219 (0.711–22.505)	0.116		
Mycophenolate mofetil	1.176 (0.226–6.127)	0.847		
Aspirin	0.308 (0.082–1.149)	0.08	0.327 (0.053–2.024)	0.229
Statins	0.333 (0.070–1.597)	0.169		
Bisphosphonate	0.967 (0.265–3.526)	0.959		
Smoking (Cigarette)	0.824 (0.174–3.903)	0.807		
Alcohol	0.833 (0.146–4.752)	0.837		
Anti-ds DNA level	With independent sample T test 78.5–111.7 (IU/ml)	0.09		

THA: total hip arthroplasty, SLE: systemic lupus erythmatosus, HCQ: hydroxychloroquine, aPL: antiphospholipid antibodies

#### Complement levels

Of the 39 cases, 29 (74.4%) cases had a normal complement levels, and 10 (25.6%) cases had a reduced complement levels. Also, four (10.3%) patients who had a low complement levels after CD did not need THA surgery and 6 (15.4%) cases needed THA surgery.

In contrast, 22 (56.4%) cases with normal complement levels did not require THA after CD surgery and 7 (17.9%) cases underwent THA (*P* < 0.04, OR: 4.71, CI 95% = 1.03–21.6). The finding indicates that complement serum levels alone can’t determine the need for THA surgery in the patients (*P* < 0.66) (Table 3).

**Table 3:** Comparison between the need for THA surgery after core decompression with investigated variables

With/without THA	Uni-variable logistic regression		Multivariable logistic regression	
	Odds ratio (CI 95%)	*P* value	Odds ratio (CI 95%)	*P* value
Male gender	0.48 (0.028–8.348)	0.614		
Bilateral Hip osteonecrosis	3.600 (0.385–33.637)	0.261		
Abnormal C3, C4 levels	4.714 (1.026–21.651)	0.046*	0.649 (0.075–5.579)	0.694
Abnormal aPL levels	4.714 (1.026–21.651)	0.046*	3.068 (0.488–19.290)	0.232
SLE relapse	9.048 (1.912–42.808)	0.005**	1.795 (0.166–19.439)	0.630
Kidney damage	2.625 (0.642–10.728)	0.179		
Neuro damage	0.458 (0.046–4.578)	0.506		
Azathioprine	14.929 (2.625–84.890)	0.002**		
HCQ	1.000 (0.206–4.856)	1.000		
Methotrexate	1.867 (0.405–8.614)	0.424		
Mycophenolate mofetil	0.764 (0.127–4.596)	0.768		
Aspirin	0.278 (0.067–1.147)	0.077	0.391 (0.063–2.409)	0.311
Statins	1.000 (0.206–4.856)	1.000		
Bisphosphonate	0.606 (0.148–2.486)	0.487		
Smoking (Cigarette)	1.260 (0.250–6.350)	0.779		
Alcohol	0.350 (0.036–3.358)	0.363		
Anti-ds DNA level	With independent sample T test 93.23–102.52 (IU/ml)	0.66		

SLE: systemic lupus erythmatosus, HCQ: hydroxychloroquine, aPL: antiphospholipid antibodies

#### Anti-phospholipid antibodies

During the follow-up after CD surgery, 29 (74.4%) cases had normal levels of anti-cardiolipin (IgM and IgG) and lupus anticoagulant (LA) antibodies, and 10 (25.6%) cases had elevated and high levels of anti-phospholipid (aPL) antibodies and had positive for LA antibody. This study indicated that the comparison between aPL levels and the need for THA surgery during follow-up was not statistically significant (*P* < 0.232, OR: 3.68, CI 95% = 0.48–19.29).

Seventeen cases (43.6%) with normal aPL levels did not show radiographic deterioration in follow-up imaging, and 12 cases (30.8%) showed some joint destruction degrees of radiographic deterioration in follow-up imaging. On the other hand, one case (2.6%) that had a high aPL levels during follow-up did not have any radiographic deterioration, and 9 cases (23.1%) had some degrees of radiographic deterioration (Table 3). Therefore, it seems that SLE patients with high aPL levels were 10.3 times more likely to have radiographic deterioration of the hip joint after CD (*P* < 0.047, OR: 10.3, CI 95% = 0.93–14.14).

### Medication consumption and THA surgery after core decompression

#### Bisphosphonate

In terms of bisphosphonate history consumption and patients satisfaction with hip joint function after CD, the five (19%) cases with a history of bisphosphonate consumption had sufficient satisfaction with their joint function, and 19 (48.7%) cases were not satisfied. In contrast, 10 (25.6%) cases (without CD surgery) with a history of bisphosphonate consumption had sufficient satisfaction with their joint function, and 5 (12.8%) cases did not have sufficient satisfaction. Using univariate logistic regression, a significant relationship between SLE with osteonecrosis who underwent CD and had a history of receiving bisphosphonate, their satisfaction with joint performance increased (86.8%) in comparison to patients with no history of bisphosphonate consumption (*P* < 0.006, OR: 0.13, CI 95% = 0.03–0.57) (Table 4). However, in radiologic assessment, out of 13 cases with radiological worsening, seven cases with a history of bisphosphonate consumption showed less radiological worsening, while eight cases exhibited radiological worsening in SLE patients (*P* < 0.91).

**Table 4:** Comparison between patient’s satisfactions from joint function after core decompression with investigated variables

Joint satisfaction	Uni-variable logistic regression		Multivariable logistic regression	
	Odds ratio (CI 95%)	*P* value	Odds ratio (CI 95%)	*P* value
Male gender	0.000 (0.000–0)	0.999		
Bilateral Hip osteonecrosis	0.585 (0.098–3.485)	0.556		
Abnormal C3, C4 levels	1346^E+12^ ( 0.000–0)	0.999		
Abnormal aPL levels	3.250 (0.586–18.034)	0.178		
SLE relapse	2.000 (0.524–7.630)	0.310		
Kidney damage	2.500 (0.666–9.385)	0.175		
Neuro damage	1346^E+12^ ( 0.000–0)	0.999		
Azathioprine	2.364 (0.619–9.023)	0.209		
HCQ	0.143 (0.016–1.288)	0.083	0.239 (0.023–2.422)	0.226
Methotrexate	1.333 (0.287–6.387)	0.719		
Mycophenolate mofetil	0.800 (0.152–4.204)	0.792		
Aspirin	0.564 (0.152–2.087)	0.391		
Statins	0.724 (0.160–3.276)	0.675		
Bisphosphonate	0.132 (0.031–0.565)	0.006**		0.02*
Smoking (Cigarette)	1.053 (0.212–5.232)	0.950		
Alcohol	3.684 (0.386–35.140)	0.257		
Anti-ds DNA level	With independent sample T test 84.77–103.55 (IU/ml)	0.35		

THA: total hip arthroplasty, SLE: systemic lupus erythmatosus, HCQ: hydroxychloroquine, aPL: antiphospholipid antibodies

#### Hydroxychloroquine (HCQ)

In this study, nine cases (23.1%) had no history of treatment with HCQ, and 30 SLE patients (76.9%) were treated with HCQ. From 30 SLE patients treated with HCQ, 1 case (2.6%) had sufficient satisfaction and 8 cases (20.5%) did not have sufficient satisfaction from their joint function after CD. In contrast, 14 cases (35.9%) who had a history of using HCQ were sufficiently satisfied with their joint function and 16 cases (41.0%) were not sufficiently satisfied with their joint function (without CD). Based on these findings, the prescription of HCQ alone, without considering other effective factors such as bisphosphonates prescription, has not significantly impacted patients satisfaction with joint function (analyzed with multivariate logistic regression model, *P* < 0.226, OR: 0.239, CI 95% = 0.023–2.422).

#### Corticosteroid

Of the 39 studied patients, the mean cumulative dose of prednisolone before and after CD surgery was 46.41 mg and 14.74 mg respectively. Six out of 23 patients discontinued the medication prednisolone completely after CD. Twenty-six hip joints did not require THA surgery with a mean prednisolone dosage of 48.26 mg, while 13 hip joints with a mean prednisolone dosage of 42.69 mg required THA surgery (*P* < 0.58). The twenty-six cases who did not need THA surgery continued prednisolone with a mean daily dose of 14.61 mg after CD, while 13 cases who underwent THA surgery continued prednisolone with a mean daily dose of 15 mg (*P* < 0.94). However, in the study did not demonstrate a statistically significant relation between the mean cumulative dose of prednisolone and probable prognostic factors (Table 5).

**Table 5:** Comparison between mean cumulative dose of prednisolone and need for THA surgery, joint radiographic deterioration with patient’s satisfaction from joint function

Mean cumulative dose of prednisolone	W/O THA	With THA	*P* value	W/O radiologic change	With radiologic change	*P* value	Joint satisfaction	Joint dissatisfaction	*P* value
Before core decompression	48.26	42.69	0.946	46.94	45.95	0.918	57.33	39.58	0.167
After core decompression	14.61	15	0.946	12.91	16.30	0.524	21.66	10.41	0.234

#### Immunosuppressive

In 19 (48.7%) cases who had no history of azathioprine consumption did not require THA, and 2 (5.1%) cases underwent THA. In contrast, 7 (26.9%) patients treated with azathioprine did not require THA and 11 (28.2%) THA operations were performed. Univariate logistic regression analysis revealed that, a statistically significant relationship between azathioprine consumption compared to require THA surgery (*P* < 0.002, OR: 14.93, CI 95% = 2.62–84.89). So, patients who take azathioprine in the course of treatment need 14.9 times more THA surgery than who do not take azathioprine in SLE patients. Of the 23 studied patients, 23.1% consumed methotrexate, and 17.9% were treated with Mycophenolate mofetil (MMF). None of the studied patients had received cyclosporine. The history of methotrexate consumption (*P* < 0.424) and MMF (*P* < 0.768) did not show a significant relationship with the need for THA surgery after CD in the current study (Table 3).

## Discussion

This study was a retrospective analysis of 23 hip SLE patients (39 affected hip joints) with osteonecrosis of the femoral head with stage II of the disease, based on the Ficat-Arlet classification system, who underwent CD. The main goal was to evaluate the prognostic factors of CD surgery in SLE patients suffering from ONFH. The factors affecting joint prognosis following CD revealed that a positive anti-phospholipid antibodies accelerates the deterioration of the joint radiograph, and the consumption of alendronate (bisphosphonate) has a significant positive effect on patient satisfaction. The cumulative steroid dose (prednisolone) and its continuing treatment following CD did not affect the patient’s prognosis.

The incidence of asymptomatic ONFH in SLE diseases is 34 to 44% [19]. In a cross-sectional study (Mont et al.,) that examined 31 hip joints with AVN in 18 patients with SLE in a period of 4 to 18 years (average 12 years), 21 joints (68%) required a total hip replacement (Table 6) [20]. In our study, out of the 39 hip joints, 33.3% of the hip joints underwent total hip arthroplasty at a minimum of 1 year and a maximum of 5 years (average of 3.5 years) from CD. The higher percentage of THA (approximately 2 times) in the Mont et al., study may be due to the longer follow-up period compared to our study.

**Table 6:** Previous studies for clinical features of patients with ONFH

Authors	Year	No. of patients with AVN	Study design	FICAT stage	Corticosteroid	THA	Conclusion
Tsai et al	2020	39	Retrospective	III & IV	95.5%	–	High daily doses of prednisolone were associated with a significant risk of AVN
Shigemura et al	2011	337	Prospective	–	51.3%	–	The incidence of corticosteroid-related to osteonecrosis varies among different underlying diseases
Maniwa et al	2000	19	Cross-sectional	I, II	66.7%	30.8%	Core decompression provides an effective treatment for steroid-associated osteonecrosis other than in cases with SLE
Mont et al	1997	50	Cross-sectional	II, III, IV	61.1%	68%	None of the factors that have been found to be related to AVN in SLE patients could be related to disease progression

In another study on 26 joints (19 patients) with osteonecrosis of the femoral head with grade I or/and II involvement, which underwent CD surgery, 17 joints (4.65%) had very good or good results with using Ficat classification. Eight joints (8.3%) required further surgery such as hip arthroplasty for 7 joints and osteotomy for 1 joint [12].

The most common aPL antibodies are the lupus anti-coagulant and anti-cardiolipin antibody. These two antibodies are often found together, but can also be detected alone in a person. The presence of these aPL antibodies in an individual is related to a predisposition for blood clots. The temporary or permanent loss of blood supply could form anywhere in the body, and it could lead to AVN. In patients with SLE, the risk of clotting does not necessarily correlate with disease activity, so the presence of these antibodies can cause problems even when a person’s SLE is under control. Many studies reported that anti-phospholipid antibodies are considered to be one of the risk factors for developing osteonecrosis [21]. In our study, patients with high aPL levels were 10.3 times more likely to develop osteonecrosis of the femoral head than patients with normal aPL levels, indicating that high levels of anti-phospholipid antibodies (particularly IgG anti-cardiolipin) play an important role in the development of AVN in SLE patients. There have been few studies on the effect of AVN risk factors on its progression in SLE patients [22–24]. In one study by Mont et al., eighteen SLE patients (31 hip joints) with AVN were compared to 48 hip joints in 32 patients with various causes of femoral head AVN. They reported that CD surgery, high levels of anti-phospholipid antibodies, and other AVN risk factors did not affect the progression of AVN deterioration in 18 SLE patients (18 cases with 31 hip joints) [20]. Another cross-sectional study of SLE patients with AVN of the femoral head indicated that the involvement severity of the articular surface of the femoral head was the most important prognostic factor of CD, while other factors like corticosteroid consumption and high levels of anti-phospholipid antibodies had no statistically significant effect on the prognosis of CD in these diseases [25]. These discrepancies may be related to the differences between study sample size, race, underlying diseases, and various causes of femoral head AVN.

A current study has explored the relationship between patients satisfaction with joint function following CD and the taking of bisphosphonate family medicines for the first time. SLE patients with ONFH who had CD surgery and a history of bisphosphonate consumption were 83.2% less dissatisfied with joint function than patients who had no consumption history of this medication (*P* < 0.02). Bisphosphonates (alendronate) have been shown in animal experiments to improve trabecular ossification and microcirculation in the femoral head, as well as to prevent corticosteroid-induced AVN of the femoral head [26]. In addition, an association between osteopenia and AVN has been shown in kidney transplant recipients [27]. There is currently no information on the protective role of bisphosphonates in humans; however, there are few evidence-based studies that bisphosphonates may play a role in ameliorative the symptoms of patients with non-femoral head AVN and delaying the onset of the disease.

In SLE disease, the most common cause of AVN is corticosteroid consumption. Although the pathogenesis of corticosteroid-related osteonecrosis is unknown, ischemia is the main cause of AVN. Some of the pathogenic processes of AVN caused by high corticosteroid regimens include fat embolism, increased intraosseous pressure (IOP) due to adipocyte hypertrophy, vasoconstriction, and decreased mesenchymal stem cells (MSC) [28]. According to a meta-analysis study, the mean daily dose, instead of the cumulative dose, was related to the severity of AVN [29]. A cross-sectional study demonstrated that in a one-year follow-up examination of the hip joint MRI, osteonecrosis was considerably higher in SLE patients than in non-SLE patients taking corticosteroids [30]. In our study, the mean daily dose of corticosteroid (oral prednisolone) before and after CD did not have a significant relationship with hip joint prognostic factors.

Other than cyclophosphamide, immunosuppressive medicines including MMF, azathioprine, and methotrexate are linked to an increased incidence of AVN in SLE patients [22]. In patients with bone marrow transplants has been demonstrated that MMF and calcineurin inhibitor consumption increases the incidence of AVN [31]. There is no evidence of these medicines effect on the prognosis of joint survival with AVN following CD. The result of the current study demonstrated that, after CD, patients who had high-dose azathioprine therapy were more likely to develop radiographic deterioration of the affected joint and the requirement for total hip replacement. However, independent of other variables impacting the study, azathioprine was unable to sustain a significant relationship with prognostic indicators. To support these findings, we can point out that azathioprine is prescribed for more severe SLE patients. The poor prognosis in azathioprine-treated patients could be attributable to the severity of the underlying disease and administration of higher doses of prednisolone; however, there is no link between azathioprine and AVN exacerbation. Finally, according to our findings and other studies, more investigation is required on the role of azathioprine in the survival of AVN joints following CD.

This retrospective cohort study had limitations. The main limitation was the lack of a control group of patients without SLE disease. Second, the sample size wasn’t great. Third, some para-clinical data were not available such as anti-beta-2-glycoproteins (β2GPI) IgG and IgM. Also, in our study, anti-dsDNA was measured using ELISA, which is more sensitive but less specific than the indirect immunofluorescence (IIF) test in SLE serum specimens. This suggests that ELISA is a more cost-effective alternative to IIF testing for initial anti-ds DNA screening. The serum specimen positive with anti-ds DNA in ELISA should be confirmed by IIF to determine the antibody titer.

Overall, evaluation of the prognostic factors in SLE patients showed that the consumption of bisphosphonates (*P* < 0.02) had a significant positive effect on patients satisfaction. Also, the presence of anti-phospholipid antibodies could (*P* < 0.047) exacerbate the deterioration of hip joint radiography.

## Conclusions

This study on the outcome of CD in SLE patients suffering from hip osteonecrosis showed that half of them had radiographic deterioration and a third of them underwent total hip arthroplasty. Based on the results, it can be concluded that performing CD in SLE patients with osteonecrosis of the femoral head (grade I and II) with high-level anti-phospholipid antibodies is not associated with a good prognosis. Also, this study did not indicate a relationship between the mean cumulative dose of corticosteroids (prednisolone) and probable prognostic factors of hip joint after CD. Taking this into account, reduction or discontinuation of corticosteroids is not recommended in these patients. However, patients who use bisphosphonate had higher satisfaction with joint function after CD surgery in this study.

## Data Availability

All data made available upon reasonable request.

## Abbreviations

SLE: Systemic lupus erythematosus
ONFH: Osteonecrosis of the femoral head
AVN: Avascular necrosis
MRI: Magnetic resonance imaging
aCL: Anti-cardiolipin antibodies
MSC: Mesenchymal stem cell
THA: Total hip arthroplasty
CD: Core decompression
IOP: Intraosseous pressure
MMF: Mycophenolate mofetil
THA: Total hip arthroplasty
HCQ: Hydroxychloroquine
aPL: Anti-phospholipid antibodies
ELISA: Enzyme-linked immunosorbent assays
